# Artificial light at night affects the daily profile of pulse pressure and protein expression in the thoracic aorta of rats

**DOI:** 10.1038/s41440-024-01685-9

**Published:** 2024-04-25

**Authors:** Hana Mauer Sutovska, Viktor Obermajer, Michal Zeman, Lubos Molcan

**Affiliations:** Department of Animal Physiology and Ethology, Faculty of Natural Sciences, Comenius University in Bratislava, Bratislava, Slovakia

**Keywords:** Artificial light at night, Thoracic aorta, Normotensive rats, Pulse pressure, Angiotensin II receptor type 1

## Abstract

Artificial light at night (ALAN) disrupts 24-h variability of blood pressure, but the molecular mechanisms underlying these effects are unknown. Therefore, we analysed the daily variability of pulse pressure, the maximum value of acceleration rate of aortic pressure (dP/dt_(max)_) measured by telemetry and protein expression in the thoracic aorta of normotensive male rats exposed to ALAN (1–2 lx) for 3 weeks. Daily, 24-h variability of pulse pressure and dP/dt_(max)_ was observed during a regular light/dark regimen with higher values during the dark compared to the light phase of the day. ALAN suppressed 24-h variability and enhanced ultradian (<12-h) periods of pulse pressure and dP/dt_(max)_ in duration-dependent manners. From beat-to-beat blood pressure variability, ALAN decreased low-frequency bands (a sympathetic marker) and had minimal effects on high-frequency bands. At the molecular level, ALAN decreased angiotensin II receptor type 1 expression and reduced 24-h variability. ALAN caused the appearance of 12-h oscillations in transforming growth factor β1 and fibulin 4. Expression of sarco/endoplasmic reticulum Ca^2+^-ATPase type 2 was increased in the middle of the light and dark phase of the day, and ALAN did not affect its daily and 12-h variability. In conclusion, ALAN suppressed 24-h variability of pulse pressure and dP/dt_(max)_, decreased the power of low-frequency bands and differentially affected the expression of specific proteins in the rat thoracic aorta. Suppressed 24-h oscillations by ALAN underline the pulsatility of individual endocrine axes with different periods, disrupting the cardiovascular control of central blood pressure.

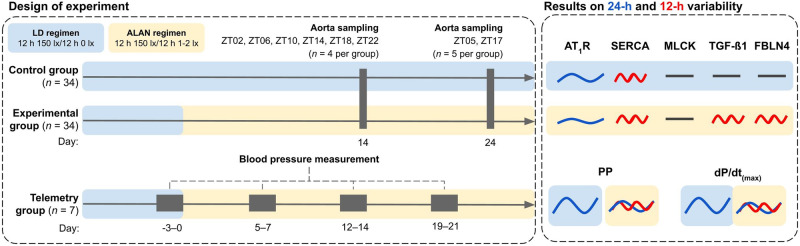

## Introduction

The biological clock generates circadian rhythms with an endogenous period of ~24-h. Circadian rhythms are observed in behaviour, locomotor activity, metabolism, and many other physiological processes, including the cardiovascular system [1–3]. The main synchronisation factor of circadian rhythms is a regular light/dark (LD) cycle. Disruption of the central circadian oscillator, the suprachiasmatic nuclei of the hypothalamus (SCN), negatively affects physiological processes, including the cardiovascular system [4]. The regular LD cycle can be disrupted by shift work, night work and artificial light at night (ALAN). Compared to shift work, which affects around 18% of people in EU countries [5] and 25% in the U.S. [6], ALAN affects about 83% of the global population, and more than 99% of EU and U.S. people live under light polluted skies for a long time during their lives [7].

Studies in humans show associations between ALAN and changes in 24-h blood pressure rhythm [8], kidney function, glomerular filtration [9] and structural changes in the blood vessels [10]. Structural changes in the vessel wall and stiffness are prominent predictors of elevated cardiovascular risk. In addition, the structural properties of vessels are involved in maintaining blood vessel compliance, thereby contributing to the regulation of pulse pressure and systolic blood pressure. Similar observations were found in animal models [11]. In rats, ALAN suppressed the 24-h variability of blood pressure and heart rate and reduced the amplitude and significance of their circadian rhythms [1]. At the same time, some individuals became arrhythmic even after 2 weeks of ALAN [12]. Distinct from humans, in rats, ALAN suppressed sympathetic activity and altered baroreflex sensitivity [1, 12], which may be associated with the observed haemodynamic changes.

The vasoactivity of arteries also depends on local endothelial factors, endocrine substances and the structure of the vessel wall [13, 14]. These variables subsequently regulate compliance, pulse pressure, pulse wave velocity, augmentation index, maximum value of acceleration rate of aortic pressure (dP/dt_(max)_) and thus, central blood pressure in large arteries. Studies with knockout models also demonstrate the significance of vascular wall composition and the presence of functional proteins in maintaining the circadian blood pressure rhythm and contractility [15–17]. Important local factors are endothelial nitric oxide synthase and endothelin-1, which are differently affected by ALAN in the thoracic aorta of rats [1]. Two weeks of ALAN increased endothelial nitric oxide synthase expression during the light phase in the rat aorta, and these effects disappeared after 5 weeks of ALAN exposure [1]. Even low light intensity at night significantly deregulates the SCN. Its output effectors include arginine vasopressin and endocrine factors [3], which are also affected by glucocorticoids, catecholamines and angiotensin II and regulate large vessels’ function [18]. Moreover, glucocorticoids and catecholamines reveal significant pulsatile secretion with an ultradian period from 50 min to 12 h [19, 20]. While the consequences of ALAN on metabolism are extensively studied, its impact on protein expression in the vasculature within a 24-h period and vascular function remains unknown. Therefore, we aimed to analyse the effects of ALAN exposure (1–2 lx) for 3 weeks on pulse pressure, dP/dt_(max)_, beat-to-beat blood pressure variability and daily protein expression variability in the thoracic aorta of normotensive male rats. We focused on the expression of proteins associated with calcium transport in cells and proteins maintaining the structure of blood vessels, which are important for maintaining the compliance of blood vessels.

## Materials and methods

### Ethical approval

This experiment was approved by the Ethical Committee for the Care and Use of Laboratory Animals at the Comenius University in Bratislava, Slovak Republic and the State Veterinary Authority of the Slovak Republic (Ro-1648/19-221/3), and in accordance with the recommendations of the ARRIVE guidelines, Guide for the Care and Use of Laboratory Animals and EU Directive 2010/63/EU for animal experiments.

### Animals

We used normotensive adult male Wistar rats from a breeding station at the Institute of Experimental Pharmacology and Toxicology, Slovak Academy of Sciences (Dobrá Voda, Slovak Republic). Animals were housed in groups of four in plastic cages in two separate rooms: control and experimental. Food and water were available *ad libitum*. The room temperature (21 ± 2 °C) and humidity (55 ± 10%) were regularly controlled. In the control room, animals were kept under a regular LD regimen of 12 h light (150 lx)/12 h dark (0 lx). In the experimental room, animals were kept under an ALAN regimen of 12 h light (150 lx)/12 h dim light (1–2 lx; dimD).

### Experimental protocol

Rats were divided into control (*n* = 34; 6.5-month-old), experimental (*n* = 34; 6.5-month-old) and telemetry-measured (*n* = 7; 4.5-month-old) groups. Control rats were housed for the whole experiment under regular 12:12 LD conditions. Experimental rats were housed under the LD regimen during the synchronisation period and were then exposed to ALAN (1–2 lx) for 14 or 24 days. Thoracic aortas without perivascular adipose tissue were immediately sampled from anaesthetised rats (4% isoflurane) after 14 days in ZT02, ZT06, ZT10, ZT14, ZT18, ZT22 of ALAN and after 24 days of ALAN in ZT05 and ZT17 (ZT00 is the beginning of the light phase). Tissue sampling during dark and dimD was done under red light. Samples were immediately snap-frozen in liquid nitrogen and stored at –80 °C for further analysis. Telemetry-implanted rats were measured in the LD regimen over 7 days and then over 24 days of ALAN.

### Haemodynamic measurement

We continuously measured dP/dt_(max)_ and pulse pressure by telemetry in freely moving rats. Telemetry sensors (HD-S10; Data Sciences International, MN, USA) were implanted in the abdominal aorta in anaesthetised (isoflurane; induction 4% in 100% oxygen; maintenance 1.5–2% in 100% oxygen) rats according to the procedures established at our department [1, 21]. Animals recovered for 2 weeks and consequently were exposed to LD and ALAN regimens, respectively. Telemetry data were measured with the frequency of 500 Hz over 5-min intervals four times per hour for 3 days at the end of LD weeks and the end of the first, second and third ALAN weeks. The frequency of blood pressure variability and dP/dt_(max)_ were calculated from the original telemetry data. We analysed the frequency of blood pressure beat-to-beat variability by absolute low-frequency (aLF) and absolute high-frequency (aHF) bands and their normalised ratio (LF/HF) [21].

### Western blot

Thoracic aortas were homogenised with a cocktail of saccharose solution, protease and phosphatase inhibitors as published previously [1, 22]. In the extracted supernatant, protein concentrations were measured using a BCA Protein Assay Kit (Thermo Fisher Scientific, Waltham, MA, USA). In aorta protein lysate, we analysed the expression of angiotensin II receptor type 1 (AT_1_R), transforming growth factor β1 (TGF-β1), sarco/endoplasmic reticulum Ca^2+^-ATPase type 2 (SERCA2), myosin light-chain kinase (MLCK) and fibulin 4 (FBLN4). Protein expressions were normalised to smooth muscle alpha-actin (αSMA). Electrophoresis separated protein lysates (30 μg) in 12% SDS-polyacrylamide gel (Owl P8DS, Owl Separation systems, USA). Separated proteins were transferred on nitrocellulose membrane and blocked in 5% bovine serum albumin in tris-buffered saline with 0.1% Tween® 20 detergent. Next, the membrane was incubated with a primary antibody (Supplementary Table 1) and subsequently with appropriate horseradish peroxidase-conjugated secondary antibody (Supplementary Table 1). After washing out, signals were detected by enhanced chemiluminescence using ClarityWestern ECL Substrate (Bio-Rad Laboratories, Hercules, CA, USA). Signals were visualised automatically on the Vü-C chemiluminescence imaging system (Pop-Bio Imaging, Milton, UK) and quantified using Quantity One Basic software (4.6.6., Bio-Rad Laboratories, Inc., USA).

### Data analysis

**Fig. 1:**
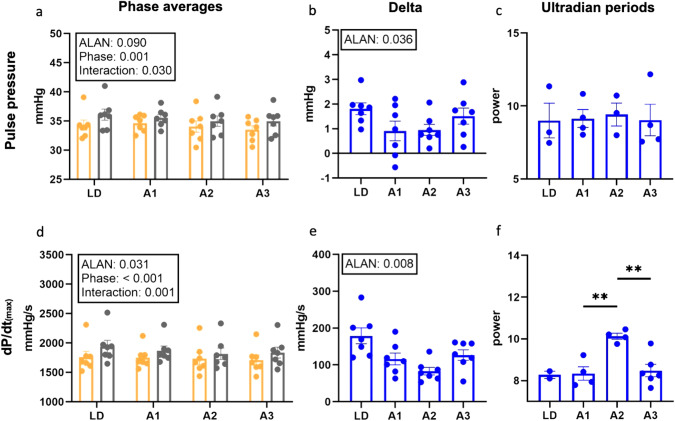
Changes in phase averages, delta (dark-light difference) and ultradian periods of pulse pressure (**a**, **b**, **c**) and maximum value of acceleration rate of aortic pressure (dP/dt_(max)_; **d**, **e**, **f**). Data are visualised as individual datapoints and arithmetic mean ± the standard error of the mean. Yellow columns, light phase of the day; grey columns, dark/dim dark phase of the day; A1, 1 week of artificial light at night (ALAN); A2, 2 weeks of ALAN; A3, 3 weeks of ALAN

**Fig. 2:**
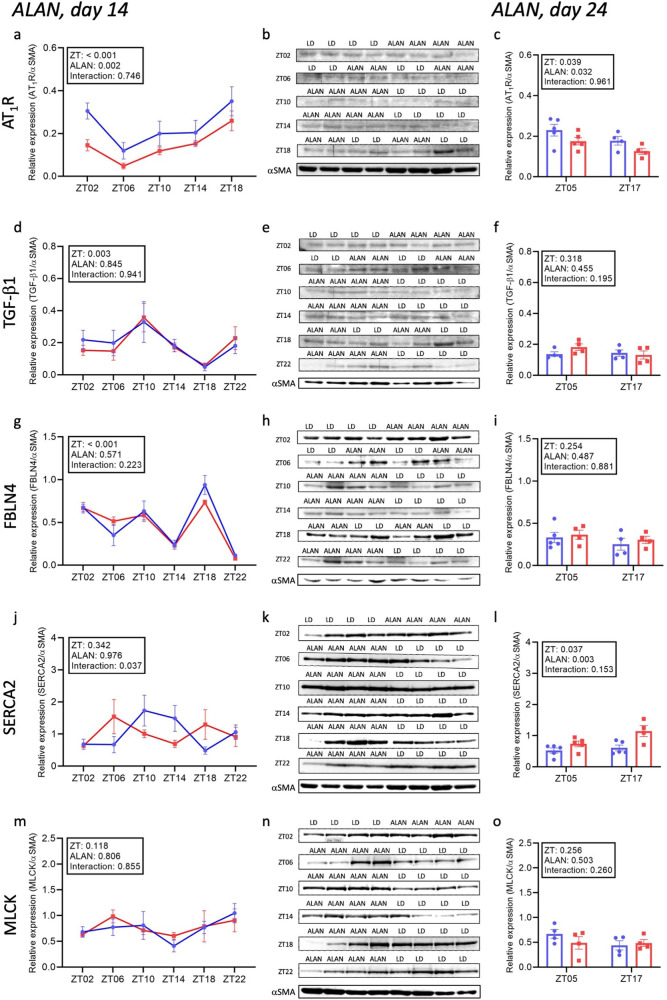
Relative protein expression of angiotensin II receptor type 1 (AT_1_R), transforming growth factor β1 (TGF-β1), fibulin 4 (FBLN4), sarco/endoplasmic reticulum Ca^2+^-ATPase type 2 (SERCA2) and myosin light-chain kinase (MLCK) in the thoracic aorta in normotensive rats exposed to 14 (**a**, **d**, **g**, **j**, **m**) and 24 days (**c**, **f**, **i**, **l**, **o**) of artificial light at night (ALAN). Representative original western blot bands (**b**, **e**, **h**, **k**, **n**) of antibody specificity in individual ZT in thoracic aorta in normotensive rats exposed to 14 days of ALAN. Data are visualised as individual datapoints and arithmetic mean ± the standard error of the mean. Blue lines and columns, control regular light/dark (LD) regimen; red lines and columns, ALAN exposure; ZT00 is the beginning of the light phase; αSMA, alpha-smooth muscle actin

**Fig. 3:**
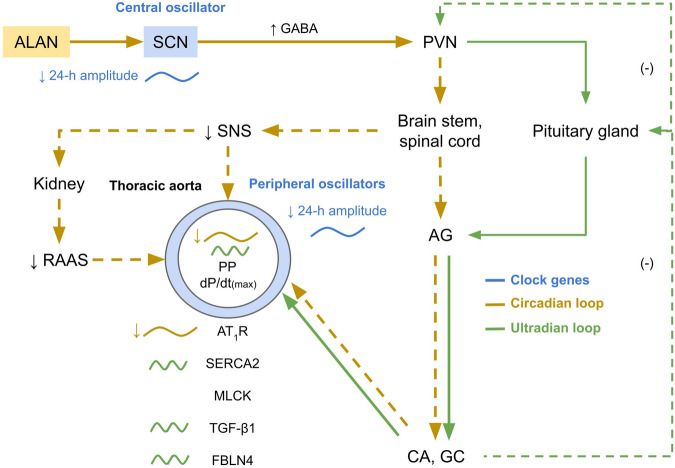
Possible pathways in how artificial light at night (ALAN) can affect thoracic aorta functions and the expression of proteins in the circadian- and ultradian-dependent manner. AG adrenal gland, AT_1_R angiotensin II receptor type 1, CA catecholamines, dP/dt_(max)_ maximum value of acceleration rate of aortic pressure, FBLN4 fibulin 4, GABA gamma-aminobutyric acid, GC glucocorticoids, MLCK myosin light-chain kinase, PP pulse pressure, PVN paraventricular nuclei, RAAS the renin-angiotensin-aldosterone system, SCN suprachiasmatic nuclei, SERCA2 sarco/endoplasmic reticulum Ca^2+^-ATPase type 2, SNS the sympathetic nervous system, TGF-β1 transforming growth factor β1

Telemetry data for pulse pressure, dP/dt_(max)_, aLF, aHF and LF/HF were compared by two-way ANOVA (factors: phase of the day, ALAN) followed by Tukey’s multiple comparisons test. Delta was calculated as the difference between the light and dark phases of the day and analysed by one-way ANOVA. Data for ultradian (less than 12 h) periods power of pulse pressure and dP/dt_(max)_ were calculated by Chronos-Fit software [23] and analysed by unpair t-test. Western blot data were analysed by two-way ANOVA (factors: Zeitgeber time [ZT00 is the onset of the light phase], ALAN) followed by Tukey’s multiple comparisons tests in GraphPad Prism 8.4.3. (GraphPad Software, California, USA). Data were visualised as the arithmetic mean ± standard error of the mean in GraphPad Prism 8.4.3 (Figs. 1–3). We analysed the presence and significance of 24- and 12-h rhythms of pulse pressure, dP/dt_(max)_ and protein expressions and their amplitudes (the difference between the peak and the mean value of a wave), acrophase (the time at which the peak of a rhythm occurs) and mesor (midline-estimating statistic of rhythm) using Cosinor2 (an R package) and CosinorOnline [24, 25]. Rhythmic parameters are expressed as the arithmetic mean [95% confidence intervals]. A *p* < 0.05 was considered statistically significant.

## Results

### Pulse pressure

During the LD regimen, pulse pressure was significantly higher (*p* < 0.001; Fig. 1a) during the dark (36 ± 1 mmHg) than the light (34 ± 1 mmHg) phase of the day. ALAN did not affect dimD pulse pressure after 1 week (36 ± 1 mmHg) but significantly decreased it after two (35 ± 1 mmHg; *p* = 0.003) and three (35 ± 1 mmHg; *p* = 0.003; Fig. 1a) weeks. ALAN decreased pulse pressure during the light phase of the day only after 3 weeks of exposure (33 ± 1 mmHg; *p* = 0.039). ALAN suppressed the delta (dark-light difference) of pulse pressure (*p* = 0.036; Fig. 1b).

During the LD week, all animals (*n* = 7) had a significant 24-h period of pulse pressure, while 12-h periods were present only in five animals (Table 1). ALAN suppressed the 24-h rhythm of pulse pressure, and rhythm was present only in 3 of 7 animals after 2 weeks of ALAN. After 3 weeks of ALAN, 24-h rhythms were again present in all animals. One week of ALAN did not affect the 12-h period but resulted in the loss of the 12-h period in 6 animals after two and 3 weeks (Table 1). Ultradian periods of pulse pressure shorter than 12 h were present in 3 of 7 animals (spectral power: 9.01 ± 1.19; Fig. 1c) during LD week. ALAN did not affect the power of ultradian periods shorter than 12 h independent from duration.

**Table 1:** Comparison of acrophase, amplitude, mesor and significance of 24-h and 12-h rhythm presence in the expression of telemetrically measured pulse pressure and maximum change in aortic pressure over time between any two sample points on the rising edge of a pressure cycle (dP/dt_max_) of rats exposed to control light/dark regime and later exposure to artificial light at night (ALAN)

		24-h period		12-h period	
		dP/dt_max_ Mean an ± SEM [count]	Pulse pressure Mean ± SEM [count]	dP/dt_max_ Mean ± SEM [count]	Pulse pressure Mean ± SEM [count]
Acrophase (h)	LD	19.58 ± 0.18 [7]	19.58 ± 0.39 [7]	[0]	8.33 ± 0.41 [5]
	A1	20.87 ± 0.25 [7] ***	15.42 ± 3.61 [7]	11.25 [1]	10.03 ± 0.19 [6]
	A2	17.73 ± 1.86 [7]	20.21 ± 0.75 [3]	2.07 ± 0.63 [2]	9.61 [1]
	A3	19.28 ± 0.39 [7]	20.24 ± 0.68 [7]	7.61 [1]	8.06 [1]
Amplitude (mmHg/s or mmHg)	LD	140.47 ± 15.06 [7]	1.26 ± 0.15 [7]	[0]	0.67 ± 0.04 [5]
	A1	117.89 ± 10.21 [7]	1.18 ± 0.18 [7]	46.31 [1]	0.81 ± 0.08 [6]
	A2	62.9 ± 6.46 [7] **	1.18 ± 0.17 [3]	42.88 ± 1.04 [2]	0.59 [1]
	A3	98.03 ± 10.44 [7] *, #	1.24 ± 0.2 [7]	48.01 [1]	0.67 [1]
Mesor (mmHg/s or mmHg)	LD	1854.95 ± 100.85 [7]	35.23 ± 0.92 [7]	[0]	34.4 ± 0.6 [5]
	A1	1820.14 ± 74.82 [7]	35.14 ± 0.54 [7]	1843.16 [1]	35.3 ± 0.65 [6]
	A2	1776.63 ± 98.42 [7] *	33.54 ± 1.13 [3]	1739.47 ± 27.02 [2]	31.72 [1]
	A3	1775.58 ± 88.58 [7] *	34.25 ± 0.78 [7]	1844.88 [1]	35 [1]

Data are expressed as the arithmetic mean ± standard error of the mean (SEM) [number of animals with a significant 24-h or 12-h period]. LD, control 12-h light and 12-h dark period; A1, A2 and A3, ALAN weeks 1–3

* *p* < 0.05 vs. LD; ** *p* < 0.01 vs. LD; *** *p* < 0.001 vs. LD; # *p* < 0.05 vs. A2

### dP/dt_(max)_

During LD week, dP/dt_(max)_ was significantly higher (*p* < 0.001; Fig. 1d) during the dark (1941 ± 105 mmHg/s) than the light (1762 ± 97 mmHg/s) phase of the day. ALAN reduced dP/dt_(max)_ during dimD already after the first week of exposure (1870 ± 78 mmHg/s; *p* < 0.01; Fig. 1d), with effects persisting during the second (1817 ± 97 mmHg/s; *p* < 0.001) and third weeks (1837 ± 92 mmHg/s; *p* < 0.001). ALAN, after 3 weeks, also decreased dP/dt_(max)_ during the light phase of the day (1710 ± 85 mmHg/s; *p* = 0.031). ALAN diminished delta of dP/dt_(max)_ (*p* = 0.008; Fig. 1e) between the dimD and light phase of the day.

All animals had a 24-h dP/dt_(max)_ period during LD week, while no animals had present 12-h periods (Table 1). ALAN decreased amplitude and mesor of a 24-h period after two weeks, and similar effects were observed after 3 weeks of ALAN. After ALAN, we observed 12-h periods only in one or two of 7 rats (Table 1). During the LD weeks, ultradian periods (<12-h) of dP/dt_(max)_ were present only in two of seven animals (spectral power: 8.28 ± 0.18; Fig. 1f). After 1 week of ALAN, ultradian periods (<12-h) were manifested in four animals with power 8.34 ± 0.32 (Fig. 1f). After 2 weeks of ALAN, power of ultradian periods (<12-h) were significantly higher (10.13 ± 0.15; *n* = 4; *p* = 0.002) than after 1 week of ALAN. After 3 weeks of ALAN, the power of ultradian periods (<12-h) decreased (8.48 ± 0.30; *p* = 0.003) compared to power after 2 weeks of ALAN, while ultradian periods were present in 6 of 7 rats.

### Blood pressure variability

During LD week, aLF were not different between the light (7.10 ± 0.50 mmHg^2^) and the dark (6.81 ± 0.37 mmHg^2^; Table 2) phase of the day. ALAN after one, two and 3 weeks decreased (*p* < 0.001) aLF during dimD (A1: 5.77 ± 0.50 mmHg^2^; A2: 6.03 ± 0.41 mmHg^2^; A3: 5.65 ± 0.28 mmHg^2^) and also during the light phase of the day (A1: 5.33 ± 0.82 mmHg^2^; A2: 6.59 ± 0.42 mmHg^2^; A3: 5.20 ± 0.66 mmHg^2^).

**Table 2:** Effects of artificial light at night (ALAN) on absolute low frequency (aLF), absolute high frequency (aHF) bands and their normalised ratio (LF/HF) of blood pressure variability

	aLF (mmHg^2^)		aHF (mmHg^2^)		LF/HF	
	light	dark	light	dark	light	dark
LD	7.10 ± 0.50	6.81 ± 0.37	3.44 ± 0.19	2.65 ± 0.18	2.20 ± 0.13	2.65 ± 0.16
A1	5.33 ± 0.82	5.77 ± 0.50	3.00 ± 0.25	2.58 ± 0.24	1.87 ± 0.13	2.30 ± 0.18
A2	6.59 ± 0.42	6.03 ± 0.41	3.25 ± 0.23	2.70 ± 0.25	2.02 ± 0.09	2.31 ± 0.15
A3	5.20 ± 0.66	5.65 ± 0.28	3.11 ± 0.26	2.72 ± 0.21	1.74 ± 0.22	2.14 ± 0.19
Phase	0.976		<0.001		0.016	
ALAN	<0.001		0.179		<0.001	
Phase x ALAN	0.017		0.050		0.638	

Data are expressed as the arithmetic mean ± standard error of the mean. LD, control 12-h light and 12-h dark period; A1, A2 and A3, artificial light at night, weeks 1–3

Absolute HF were significantly higher (*p* < 0.001) during the light (3.44 ± 0.19 mmHg^2^) than the dark (2.65 ± 0.18 mmHg^2^; Table 2) phase of the day. ALAN did not affect aHF during the dimD. However, ALAN suppressed aHF during the light phase of the day after one (3.00 ± 0.25 mmHg^2^; *p* = 0.008) and 3 weeks of exposure (3.11 ± 0.26 mmHg^2^; *p* = 0.067).

Ratio LF/HF were significantly higher (*p* = 0.005; Table 2) during the dark (2.65 ± 0.16) than the light (2.20 ± 0.13) phase of the day in LD week. ALAN suppressed LF/HF during dimD after one (2.30 ± 0.18; *p* = 0.038), two (2.31 ± 0.15; *p* = 0.042) and 3 weeks (2.14 ± 0.19; *p* = 0.001) of exposure. During the light phase of the day, ALAN suppressed LF/HF after one (1.87 ± 0.13; *p* = 0.046) and three (1.74 ± 0.22; *p* = 0.003) weeks of exposure.

### Protein expressions

During control LD, AT_1_R significantly differed between light and dark phases of the day if we compared five time points within a day (*p* < 0.001; Fig. 2a) and in the middle of light and dark (*p* = 0.039; Fig. 2c). Similarly, cosinor analysis showed significant 24-h (*p* = 0.024) but not 12-h (*p* = 0.796) variability in AT_1_R expression (Supplementary Table 2). ALAN decreased AT_1_R expression after 14 (*p* = 0.002; Fig. 2a) and 24 days (*p* = 0.032; Fig. 2c). Moreover, cosinor analysis showed significantly decreased mesor (from 0.258 [CI: 0.209–0.308] to 0.159 [CI: 0.134–0.184]) and decreased 24-h amplitude (from 0.119 [CI: 0.042–0.196] AT_1_R/αSMA to 0.095 [CI: 0.059–0.131] AT_1_R/αSMA) and phase-advanced acrophase (from 20.76 h [CI: 18.74–22.79 h] to 19.22 h [CI: 17.82–20.63 h]).

Expression of TGF-β1 changed over time (Fig. 2d; *p* = 0.003) during a regular LD regimen, but 24- and 12-h variability was not present (Supplementary Table 2). Moreover, we did not observe a difference in TGF-β1 by comparing the middle of the light and dark phases of the day (Fig. 2f). ALAN did not affect TGF-β1 expression after 14 (Fig. 2d) and 24 days (Fig. 2f) of exposure. On the other hand, we observed significant (*p* = 0.009) 12-h variability (Supplementary Table 2) after ALAN exposure.

Expression of FBLN4 did not differ (Fig. 2i) between the middle of the light and dark phases of the day during the regular LD regimen when the analysis was done from two time points. However, the expression of FBLN4 significantly changed over time (Fig. 2g; *p* < 0.001), but 24- and 12-h variability was not significant (Supplementary Table 2). ALAN did not affect the expression of FBLN4 after 14 (Fig. 2g) and 24 days (Fig. 2i) of exposure. On the other hand, 12-h variability of FBLN4 after ALAN reached the level *p* = 0.054.

In regular LD, SERCA2 expression did not change during 24-h (Fig. 2j), but we observed a significant difference in SERCA2 (Fig. 2l; *p* = 0.037) by comparing the middle of the light and dark phases. Cosinor analysis revealed almost significant 12-h (*p* = 0.059), not 24-h (*p* = 0.107) variability (Supplementary Table 2). After 14 and 24 days of ALAN, SERCA2 expression increased (Fig. 2l; *p* = 0.004) in the middle of the light and dark phases of the day. Overall, 24-h variability was not affected by ALAN. In contrast, the amplitude of 12-h variability slightly decreased (p = 0.060; from 0.476 [CI: 0.110–0.843] SERCA2/αSMA to 0.439 [CI: 0.100–0.780] SERCA2/αSMA) and acrophase was significantly advanced from 11.267 h (CI: 9.798–12.736 h) to 6.764 h (5.288–8.239 h; Supplementary Table 2).

MLCK expression did not oscillate in the regular LD regimen when analysed from six (Fig. 2m) and two time points (Fig. 2o) within a day. Similarly, cosinor analysis did not reveal significant 24- and 12-h variability (Supplementary Table 2). ALAN did not affect MLCK expression after 14 (Fig. 2m) nor after 24 days (Fig. 2o) and 24- and 12-h variability (Supplementary Table 2).

## Discussion

The impact of ALAN on blood pressure and heart rate depends on the duration of exposure, with the most pronounced changes manifesting after 2 weeks of ALAN exposure. These effects are likely mediated through the autonomic nervous system, involving transmission from the SCN to the heart and vasculature [1, 22]. Although the effects of ALAN on blood pressure and its rhythm are known from previous studies [1, 12], the effects of ALAN on the functionality and structure of blood vessels remain unknown or are often limited to a one-time point or in the light phase of the day. In addition to small resistance vessels, large conduit vessels, such as the thoracic aorta, play an important role in maintaining haemodynamics. We evaluated the functional properties of blood vessels by the telemetry measurement of pulse pressure and dP/dt_(max)_ and by expression of selected proteins in the thoracic aorta. From the telemetry data, we estimated circadian (24-h) and ultradian (<12-h) periods. Using blood pressure beat-to-beat variability, we evaluated the influence of the autonomic nervous system on blood vessels. Further, we analysed the daily and 12-h variability of selected proteins in the thoracic aorta of normotensive rats exposed to ALAN for 14 or 24 days.

Like heart rate and blood pressure [1, 12], pulse pressure and dP/dt_(max)_ were elevated during the dark time and showed significant 24-h rhythms in rats kept at the LD regime. ALAN reduced the pulse pressure difference between the light and dark phase of the day and suppressed the 24-h variability, which was caused by the reduced pulse pressure during the dimD phase of the day. The observed effects depended on the length of ALAN exposure; the most significant effects were observed after 2 weeks of ALAN. Similar results were observed in the case of decreased systolic blood pressure, heart rate and sympathetic nervous activity in normotensive Wistar rats [1, 12, 22] but with a delay in spontaneously hypertensive rats [2] and rats exposed to prenatal hypoxia, which have naturally increased sympathetic activity [12]. In the present study, we calculated sympathetic and vagal activity from blood pressure variability and aLF, aHF indices and their ratio. Concerning aLF, which reflects sympathetic nervous activity, we did not notice a difference between the phases of the day. In contrast, aHF exhibited higher activity levels during the light phase of the day under the regular LD regimen. ALAN caused suppression of aLF activity during the light and dark phases of the day and, conversely, tended to reduce aHF activity during the light phase of the day.

The sympathetic nervous system transmits photoperiodic information from the central circadian oscillator to the periphery (Fig. 3) and modulates the renin-angiotensin-aldosterone activity. In our study, we measured the protein expression of AT_1_R that binds angiotensin II whose synthesis is under circadian control [26]. The cosinor analysis revealed the presence of a 24-h oscillation in AT_1_R expression, with the lowest expression observed at the light-to-dark phase transition in regular lighting conditions. A similar profile was also demonstrated at the AT_1_R mRNA expression level in the aorta of Wistar-Kyoto rats [27]. Similar to aLF, AT_1_R expression was suppressed in both the light and dimD phases after ALAN exposure. The decrease in AT_1_R and reduced sympathetic nerve activity can partially explain the decline in blood pressure observed in rats exposed to ALAN [1, 22]. The relationship between the circadian system and AT_1_R was shown in knockout mice since deletion of *Per2* decreased the expression of the AT_1_R [28]. Similarly, suppressed or phase-advanced expression of *Per2* was observed in the SCN [3] and peripheral oscillators in the liver, spleen and adipose tissue of normotensive rats [3, 29]. In addition, *Per2* deletion inhibited signal transduction in the renin-angiotensin-aldosterone system [28]. In our experiment, ALAN (14 and 24 days) significantly suppressed the expression of AT_1_R. It is in accordance with the hypothesis that the expression of AT_1_R is directly controlled by *Per2* [25]. The autonomic nervous regulation analysis findings also indicate the sympathetic system’s involvement. It is further related to a decrease in the expression of AT_1_R in the heart’s left ventricle in normotensive rats following exposure to ALAN. This decrease is associated with reduced sympathetic activity and enhanced lusitropic effects [22]. Our data suggests that ALAN comprehensively regulates the cardiovascular system through the endocrine and autonomic nervous systems.

Low pulse pressure is associated with changes in the structure and function of blood vessels, impacting arterial compliance and arterial ability to contract and dilate effectively. However, reduced pulse pressure may reflect changes at the vascular level, insufficient heart contractility and reduced stroke volume, indicating heart failure. Reduced pulse pressure can impair haemodynamics and inadequate blood flow to vital organs [30]. The decrease in contractility during ALAN can be indirectly derived from the decrease in dP/dt_(max)_, whose 24-h amplitude and mesor were reduced after ALAN exposure. These effects were most dominant after 2 weeks of ALAN. On the other hand, after 3 weeks of ALAN, we observed a partial restoration of 24-h variability, corresponding with the heart rate of rats exposed to ALAN [1]. In contrast with a decrease in 24-h variability, we observed an increase in ultradian oscillations after ALAN exposure. Elevated power of ultradian oscillations of several physiological parameters, such as systolic blood pressure, heart rate and sleep characteristics, was recorded in different models of circadian disruption, such as shift work, constant light and jet lag [31–33]. Ultradian oscillations in the energy metabolisms became more pronounced after SCN lesions and the deletion of clock genes [34–37].

Clinical studies have demonstrated that individuals with impaired autonomic regulation of blood pressure frequently exhibit a reduction in pulse pressure [38, 39]. Furthermore, animals with lowered pulse pressure have shown a decrease in matrix metalloproteinase-2 expression [40]. Another study observed reduced vascular matrix metalloproteinase-9 expression in animals with decreased dP/dt_(max)_ [41]. Therefore, we analysed the effect of ALAN on structural and functional proteins expressed in the thoracic aorta, which can lead to a change in hemodynamics. Cosinor analysis revealed 24-h oscillations in the expression of TGF-β1 and FBLN4 in a regular LD regimen, indicating that these proteins are probably not under circadian control. FBLN4 is important for the proper integrity of blood vessels and the formation of elastic fibres, and TGF-β1 is a key factor involved in tissue remodelling. Their activation leads to fibrosis and causes a loss of vessel elasticity, which is related to a shift in the ratio of collagens and elastin [42]. In our experiment, ALAN (14 and 24 days) had no effects on total FBLN4 and TGF-β1 expression, meaning that short-term ALAN exposure did not result in structural changes in the thoracic aorta in rats. Changes in the 24-h blood pressure rhythm due to ALAN did not necessarily have to be associated with vessel structural changes. Independence of blood pressure changes from the structural properties of blood vessels was observed in patients with a non-dipping blood pressure profile, which is unrelated to arterial stiffness [43]. It agrees with our results because we did not observe changes in structural proteins at the total expression level despite the effect of ALAN on pulse pressure and dP/dt_(max)_. On the other hand, we cannot exclude the possibility that prolonged exposure to ALAN might also affect the structural proteins as a compensatory consequence of pressure changes in the blood vessel during the 24-h cycle.

Although TGF-β1 and FBLN4 did not exhibit 24-h variability in the LD regimen, we observed enhanced 12-h oscillations in FBLN4 and TGF-β1 after ALAN exposure. Therefore, we hypothesise that suppressed circadian control by ALAN can underline SCN-independent physiological rhythms, such as the pulsatile release of catecholamines and glucocorticoids (Fig. 3), with periods from 50 min to 12 h [19, 44], which was also reflected in highlighting the 12-h oscillation of pulse pressure and dP/dt_(max)_.

In the case of MLCK and SERCA2, we observed almost significant 12-h but not 24-h oscillations. SERCA2 activity is regulated by several neurohumoral factors, including catecholamines [45] and glucocorticoids [46], which are released from the adrenal glands in ultradian pulses [47] and thus, can contribute to the 12-h oscillations (Fig. 3). Under normal physiological conditions, it is possible that the expression of calcium-regulating proteins does not change significantly during the day and is influenced by the prolonged action of external factors, for example, ALAN. In our study, ALAN (24 days) increased the expression of SERCA2 in the middle of the light and dimD phases of the day. We observed a similar pattern after 14 days of ALAN when we analysed six time points. In arteries, increased expression of SERCA2 elevated the calcium pool in the sarcoplasmic reticulum, which is necessary for an increased contractile response. This can explain the surprisingly exaggerated pressure response to norepinephrine in rats exposed to ALAN [1, 48]. It’s important to note that chronodisruption can adversely affect both nocturnal and diurnal animals and humans. The circadian rhythm is essential for the correct timing of processes in the cardiovascular system. Experimental and clinical research showed that ALAN not only increases stress sensitivity and vulnerability in both rats [21] and humans [49] but also leads to the development of cardiometabolic diseases [2, 50]. In our works, ALAN decreased the robustness and amplitude of the circadian rhythm in the blood pressure and altered protein expression in the aorta, which can represent a risk for the development of cardiometabolic diseases and an attenuated ability to anticipate load. However, further studies are needed to understand the effects of ALAN on cardiometabolic health in animals and humans.

Limitations: We are unable to determine whether the observed protein expression in the thoracic aorta after ALAN is the cause of a changed daily variability of haemodynamic parameters or whether their changed light-to-night variability is the cause of changes in blood haemodynamics, which may result in a difference in the expression of proteins in the aorta. Due to technical reasons, we performed sampling only at two-time points in the second experiment (ALAN, 24 days). Six samples in 24 h do not allow us to comment on oscillations other than 24- and 12-h. In our experiment, we used normotensive rats with significant 24-h blood pressure rhythm. However, it would be interesting to measure protein expressions in rats with hypertension due to increased sympathetic activity [2, 12] or angiotensin II [51], which could lead to distinct responses of rats to ALAN.

## Conclusion

ALAN exposure (1–2 lx) for 3 weeks affected pulse pressure, dP/dt_(max)_ and protein expression in the thoracic aorta with 24-h and 12-h periods. ALAN reduced pulse pressure and dP/dt_(max)_, their differences between light and dimD phase of the day and suppressed 24-h oscillations, thereby unveiling their 12-h oscillations. Effects of ALAN on haemodynamics were duration-dependent, and the most pronounced effects were observed during the dimD phase, but ALAN also had consequences in the light phase of the day. Regarding autonomic nervous regulation, ALAN reduced sympathetic activity and had minimal impacts on parasympathetic activity. At the molecular level, ALAN reduced the mesor and amplitude of the 24-h rhythm in AT_1_R expression. In TGF-β1 and FBLN4, ALAN reinforced 12-h oscillations, which were not manifested in LD. SERCA2 showed 12-h but not 24-h variability after ALAN and in LD. Moreover, ALAN increased SERCA2 expression in the middle of light and dimD phases. Circadian disruption and associated suppression of daily variability of pulse pressure, aLF and functional-morphological changes of blood vessels may indicate an impaired ability of the cardiovascular system to anticipate load and response to stress.

## Supplementary information

Supplementary information

## Data Availability

Data will be made available on request.
