# Supplementary material for: Artificial light at night affects the daily profile of pulse pressure and protein expression in the thoracic aorta of rats
Source: Hypertens Res. 2024 Apr 25;47(7):1897–907. doi: 10.1038/s41440-024-01685-9 (PMC11224016; doi:10.1038/s41440-024-01685-9)
Supplement: Supplementary file 1 — Supplementary information [file 41440_2024_1685_MOESM1_ESM.docx]

# Supplementary

**Supplementary Table 1.** The primary and secondary antibodies used in Western blot analysis.

| antibody type | name | host species | dilution | product code | manufacture | predicted/observed MW [kDa] |
| --- | --- | --- | --- | --- | --- | --- |
| primary | **AT_1_R** | rabbit | 1:1000 | sc-1173 | Santa Cruz | 43/43 kDa |
| primary | **TGF-b1** | rabbit | 1:1000 | ab215715 | Abcam | 44/44 kDa |
| primary | **FBLN4** | rabbit | 1:1000 | bs-13059R | Bioss | 47/47 kDa |
| primary | **SERCA2** | mouse/rabbit | 1:5000 | ab2861/ab150435 | Abcam | 115/97 kDa |
| primary | **MLCK** | rabbit | 1:5000 | ab76092 | Abcam | 130/130 kDa |
| primary | **αSMA** | mouse | 1:10000 | A2547 | Sigma-Aldrich | 42/42 kDa |
| secondary | **anti-mouse** | horse | 1:1000-1:5000 | 7076 | Cell Signaling |  |
| secondary | **anti-rabbit** | goat | 1:1000-1:5000 | 7074 | Cell Signaling |  |

αSMA, alpha smooth muscle actin; AT_1_R, angiotensin II receptor type 1; FBLN4, fibulin 4; MLCK, myosin light-chain kinase; MW, molecular weight; SERCA2, sarco/endoplasmic reticulum Ca^2+^-ATPase type 2; TGF-β1, transforming growth factor β1.

**Supplementary Table 2:** Comparison of acrophase, amplitude, mesor and significance of 24-h and 12-h rhythm presence in the expression of selected proteins in the thoracic aorta of control rats and after exposure to artificial light at night (ALAN). Data are expressed as the arithmetic mean [95% confidence intervals].

| Period | Group | Parameter | AT_1_R | FBLN4 | MLCK | SERCA2 | TGF-β1 |
| --- | --- | --- | --- | --- | --- | --- | --- |
| 24 | **Control** | Acrophase (h) | 20.764 [18.736, 22.793] | 15.813 [-0.770, 32.396] | 0.198 [-5.976, 6.420] | 11.641 [8.286, 14.997] | 7.343 [4.083, 10.603] |
|  |  | Amplitude | 0.119 [0.042, 0.196] | 0.045 [-0.149, 0.239] | 0.147 [-0.054, 0.348] | 0.429 [0.052, 0.806] | 0.093 [0.011, 0.176] |
|  |  | Mesor | 0.258 [0.209, 0.308] | 0.491 [0.353, 0.628] | 0.748 [0.606, 0.890] | 1.019 [0.753, 1.286] | 0.196 [0.139, 0.254] |
|  |  | P-value | 0.024 | 0.904 | 0.375 | 0.107 | 0.111 |
|  | **ALAN** | Acrophase (h) | 19.224 [17.816, 20.632] | 6.870 [0.544, 13.196] | 1.983 [-8.047, 11.919] | 7.786 [-7.085, 22.657] | 8.747 [4.306, 13.188] |
|  |  | Amplitude | 0.095 [0.059, 0.131] | 0.087 [-0.057, 0.230] | 0.074 [-0.119, 0.267] | 0.099 [-0.287, 0.485] | 0.064[-0.011, 0.139] |
|  |  | Mesor | 0.159 [0.134, 0.184] | 0.468 [0.366, 0.570] | 0.772 [0.636, 0.909] | 1.014 [0.741, 1.286] | 0.186 [0.133, 0.239] |
|  |  | P-value | 0.000 | 0.508 | 0.756 | 0.882 | 0.264 |
| 12 | **Control** | Acrophase (h) | 3.300 [-1.323, 7.923] | 5.421 [3.251, 7.590] | 8.810 [7.188, 10.432] | 11.267 [9.798, 12.736] | 11.252 [9.365, 13.139] |
|  |  | Amplitude | 0.032 [-0.062, 0.126] | 0.161 [-0.022, 0.343] | 0.222 [0.033, 0.410] | 0.476 [0.110, 0.843] | 0.083 [-0.002, 0.167] |
|  |  | Mesor | 0.230 [0.169, 0.291] | 0.491 [0.362, 0.620] | 0.748 [0.615, 0.881] | 1.019 [0.760, 1.278] | 0.189 [0.131, 0.248] |
|  |  | P-value | 0.796 | 0.249 | 0.093 | 0.059 | 0.184 |
|  | **ALAN** | Acrophase (h) | 4.252 [-0.104, 8.608] | 5.211 [3.766, 6.655] | 7.456 [5.270, 9.642] | 6.764 [5.288, 8.239] | 10.593 [9.506, 11.680] |
|  |  | Amplitude | 0.022 [-0.042, 0.086] | 0.171 [0.042, 0.300] | 0.160 [-0.023, 0.343] | 0.439 [0.100, 0.780] | 0.112 [0.048, 0.176] |
|  |  | Mesor | 0.140 [0.010, 0.181] | 0.468 [0.377, 0.559] | 0.772 [0.643, 0.902] | 1.014 [0.774, 1.253] | 0.186 [0.141, 0.231] |
|  |  | P-value | 0.800 | 0.054 | 0.254 | 0.060 | 0.009 |

AT_1_R, angiotensin II receptor type 1; FBLN4, fibulin 4; MLCK, myosin light-chain kinase; SERCA2, sarco/endoplasmic reticulum Ca^2+^-ATPase type 2; TGF-β1, transforming growth factor β1.
